# How Bad is it? Elite Influence and the Perceived Seriousness of the Coronavirus Pandemic

**DOI:** 10.1017/XPS.2020.45

**Published:** 2020-12-18

**Authors:** Philip Moniz

**Affiliations:** Department of Government, University of Texas, Austin, TX, USA, Twitter: @MilipPhoniz

**Keywords:** Coronavirus, elite influence, perceived problem seriousness, motivated reasoning, public health, democratic accountability

## Abstract

In spite of its immense global impact, Republicans and Democrats disagree on how serious a problem the coronavirus pandemic is. One likely reason is the political elites to whom partisans listen. As a means of shoring up support, President Trump largely downplayed but at times hyped the severity of the virus. Do these messages influence the perceived seriousness of the virus’s death toll, how the president is evaluated as well as support for and compliance with social distancing guidelines? Results suggest that Republican identifiers had by early June crystallized their views on the virus’s seriousness, the president’s performance, and social distancing policies and behaviors. Unexpectedly, information critical of President Trump’s policy decisions produced a backlash causing people to show less concern about the virus’s death toll and rate the president’s performance even more highly.

## Introduction

Political elites try to bolster their support by shaping citizens’ perceptions of reality.^1^ During normal times, this is a concern for the functioning of democracy; during a period of crisis, such as the coronavirus pandemic, it may mean the difference between life and death. Despite the pandemic’s scale, people, divided by party, disagree on the severity of the problem (Mitchell and Oliphant 2020; Rothwell 2020). This paper explores the malleability and consequences of the variability of people’s sense of seriousness of the coronavirus pandemic.

Why might elites want to shape citizens’ perceptions of the seriousness of problems besetting society? One reason is to influence how citizens will evaluate them. During the coronavirus pandemic, President Trump and other political elites have repeatedly praised the job they’ve done while also downplaying the number of deaths the virus has claimed. At the same time, Trump has cast himself as a wartime president facing unprecedented adversity. Have these attempts been successful? The answers to these questions have far-reaching implications for representative democracy and accountability. If politicians can influence how they are assessed, even if their performance is poor, then democratic accountability is seriously called into question. Influencing people’s sense of how severe the crisis is may have tangible effects on their personal behavior as well, thus affecting public health. By downplaying the virus, President Trump may have hampered support and compliance with vital social distancing measures.

## Elite influence and the perceived seriousness of the coronavirus death toll

While elite influence on partisans’ opinions is well known (Zaller 1992), influence on their perceptions of reality is only beginning to be understood (Bisgaard and Slothuus 2018b). In the realm of democratic accountability, these perceptions are integral. Citizens must look back – accurately – and judge the incumbent’s performance in relation to some standard or benchmark. Research on economic voting shows that voters appear to use other countries’ economic performances as such a benchmark (Kayser and Peress 2012). Without a benchmark, it is difficult for voters to know what counts as “good” performance and what counts as “bad” (Olsen 2017). This fact may make it easier for elites to manipulate people’s perceptions (Rudisill 2013). During the pandemic, for instance, President Trump has argued that the projected death toll reflects a “very good job” in comparison with projections of worst-case scenarios (McGraw 2020). With a less familiar issue, such as the novel coronavirus, it may be even easier for elites to manipulate public sentiment because people do not know how to choose the relevant benchmarks (e.g. Rudisill 2013).

During the early months of the pandemic, the Trump administration used almost daily press briefings to help establish a preferred benchmark, from comparing the coronavirus death toll to that from automobile accidents, seasonal flu, and worst-case epidemiological projections. These are attempts to persuade the public that the number of Americans dying from COVID-19 is not very alarming compared with what it could have been absent from any governmental action.

Previous research has found that political leaders can affect how seriously people perceive a problem to be, especially among their partisans (Bisgaard and Slothuus 2018a). This is an important finding, but it remains to be seen whether this effect holds for other problems and whether it has downstream effects on other variables, such as performance evaluation and personal behavior (e.g. Bisgaard 2015; Peterson and Simonovits 2018).

Second, there is the question of whether additional information can reduce the effect of elite influence. While research shows that more information can help counteract the effect of elite cues (Bullock 2011), we do not know what type of information is most effective in doing this. Information about politicians’ past policy decisions should be instructive here (Ashworth, Bueno de Mesquita, and Friedenberg 2018). In the case of COVID-19, many political elites were not content to watch the president tell his own story without adding in counterpoints about his administration’s policies toward public health funding and preparation. Therefore, it is crucial to test the robustness of elite influence on perceptions in the face of counterarguments.

## Hypotheses

In light of these considerations, I conducted a survey experiment in which Republican subjects were exposed to elite messaging and regarding the seriousness of the coronavirus pandemic’s death toll. Messaging that makes the problem appear more serious is *problem-amplifying*, while *problem-minimizing* messaging downplays its seriousness. President Trump attempted to downplay the virus’s public health impact on several occasions by comparing the death toll to what it could have been without strict social distancing, and even to automobile fatalities and the seasonal flu (Brooks 2020; McGraw 2020). While not as often, the president also tried to amplify the seriousness of the problem, at times by comparing it to a war and casting himself as a wartime president.

### Perceived problem seriousness

Given the novelty of the coronavirus and the strength of partisan-motivated reasoning, partisans’ perceptions of the severity of the crisis should respond positively to the president’s rhetoric (Bolsen, Druckman, and Cook 2014).

Hypothesis 1 President Trump’s problem-minimizing (problem-amplifying) rhetoric with regard to the COVID-19 death toll will decrease (increase) its perceived seriousness among his partisans.

### Trump performance evaluation

By comparing the death toll from COVID-19 to its worst-case scenario, President Trump also aimed to improve his performance evaluations. On April 29, with nearly 60,000 dead, the president compared the death toll to the 2.2 million projection, and even as late as May 26, with over 100,000 dead, the president compared it with 1.5–2 million possible deaths. If the source is trusted and the information seems credible, that benchmark could stick and help raise evaluations of his administration’s performance.

Hypothesis 2 President Trump’s problem-minimizing rhetoric will increase his pandemic-specific job approval.

To further shape the public’s evaluation of his performance, President Trump emphasized his administration’s response, not its preparedness. Criticizing the federal government’s preparedness has also been a way to shift blame onto his predecessors (Jacobs and Langreth 2020). This strategy may work as voters also appear to give less weight to preparedness than to reaction during disasters (Healy and Malhotra 2009). Part of this bias may be due to people not knowing what a better prepared government and response would have looked like. During the pandemic, for instance, President Trump was criticized for dissolving the “pandemic task force” in 2018, which had been established within the National Security Council by President Obama after the Ebola crisis (Cameron 2020). This observation is important because it exemplifies how the president’s public health policy did not prioritize preparedness.

Hypothesis 3 Learning about Trump’s policy decisions to reduce the government’s pandemic preparedness counteracts the increase in his performance evaluation he received from employing problem-minimizing rhetoric.

Note that Hypothesis 3 requires Trump’s performance evaluation to have increased in the Problem-Minimizing condition in comparison with the control, and that the reference group for Hypothesis 3 is the Problem-Minimizing condition. This is what is meant by saying that the Problem-Minimizing with Counterargument treatment “counteracts” the effect of the Problem-Minimizing treatment.

### Social distancing support and intentions

A major concern for public health efforts is that people who pay attention to elites who try to downplay the virus will also ignore measures meant to mitigate its spread. If, however, people do not consider their personal risk to the virus to be very high, they should be more likely to engage in risky behaviors (Rudisill 2013). Conversely, if the severity of the virus is amplified, people should become more likely to support and engage in social distancing behaviors.

Hypothesis 4 President Trump’s problem-minimizing (problem-amplifying) rhetoric with regard to the COVID-19 death toll will decrease (increase) support for and intention to comply with social distancing measures.

## Procedure and measurement

Survey participants (N = 1,615, 46% female) were pre-screened Republican identifiers (including leaners) recruited through Prolific and were paid $1.15 to complete the survey between June 4 and 9, 2020. After giving informed consent, subjects were randomly assigned to read one of the three presentations of President Trump’s problem-manipulating rhetoric; the fourth, control group read that US deaths from COVID-19 had just passed 100,000. In the Problem-Minimizing condition, the president says that the death toll could have been over 1.5 million had he not responded the way he did; in the Problem-Minimizing with Counterargument condition, his statement is paired with an excerpt from a news article on the disbandment of the White House’s pandemic task force in 2018; and in the Problem-Amplifying condition, the president says the deaths from COVID-19 have been “a terrible thing.” After the stimulus, subjects answered the problem item, job performance scale, policy support scale, behavioral intention scale, a manipulation check, political knowledge/interest items, and demographics.

All dependent variables were scaled to take values between 0 and 1. Perceived seriousness of the COVID-19 death toll was measured on a 5-point scale asking about the size of the problem (e.g. “a very big problem” to “not a problem at all”). Evaluation of President Trump’s pandemic-specific job performance was measured by averaging together four items (e.g. “good” to “poor”) into a scale (Cronbach’s *α* = 0.97). Policy support (e.g. “requiring most businesses other than groceries and pharmacies to close”) is an 8-item scale (Cronbach’s *α* = 0.92), and intentions to comply with social distancing (e.g. “fly on an airplane”) is also an 8-item scale (Cronbach’s *α* = 0.90). Higher values correspond to greater seriousness, job performance, support, and intention. Item wording of stimuli and items can be found in the supplementary materials. All of the measurements, hypotheses, and analytic procedures follow how the plan in the registered report, which was deposited in a public repository before data collection. The registered report can be found at https://osf.io/kjh9b.

## Results

**Figure 1:**
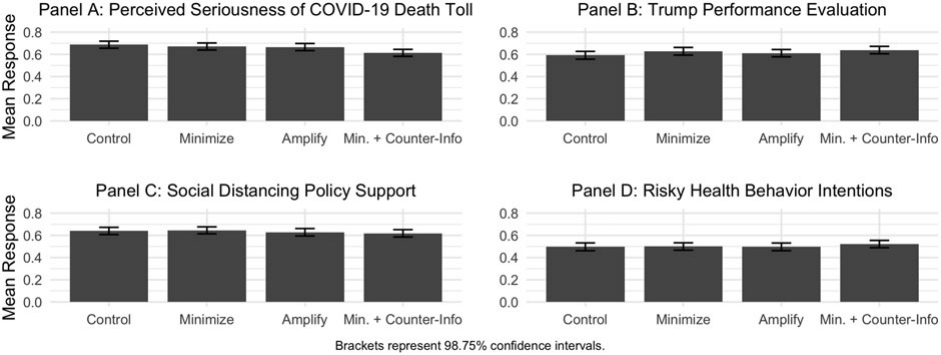
Experimental Results.

Subjects who read rhetoric President Trump’s statements either downplaying or amplifying the seriousness of the coronavirus’s death toll found it no more or less serious than subjects in the control group (see Figure 1, panel A). The average perceived seriousness decreased slightly in the Problem-Minimizing condition (*p* = 0.18, one-tailed), and it actually decreased more in the Problem-Amplifying condition (*p* = 0.12, one-tailed), contrary to expectations. The Bayes factor^2^ in favor of the alternative hypothesis is fairly small (*BF*_10_ = 0.19), meaning that the evidence is about five times more likely under the null (Held and Ott 2018). These results provide no direct support for Hypothesis 1.

The Problem-Minimizing condition raised the president’s pandemic-specific job performance by 3.6% points, but it is not statistically significant at the Bonferroni-corrected *α* = 0.0125 (*p* = 0.03, one-tailed), (*BF*_10_ = 0.19). The result provides weak to no direct evidence of a positive effect of the president’s problem-minimizing rhetoric on his job performance (Hypothesis 2). The effect of the problem-amplifying rhetoric is far from significant (*p* > 0.2, one-tailed).

Hypothesis 3 proposed that criticism of President Trump’s pandemic-specific job performance would counteract the positive effect of his self-promoting rhetoric. The counterargument did not reduce the president’s performance evaluations compared to the Problem-Minimizing treatment without counterargument (*p* > 0.2, one-tailed). Contrary to expectations, subjects rated the president’s job performance 4.7% points *better* in the Problem-Minimizing with Counterargument condition as compared to the control (*p* < 0.01, one-tailed).

The estimated effects of the president’s rhetoric on citizens’ support for and intention to comply with social -distancing guidelines were statistically nonsignificant (*p*’s > 0.50, *BF*_10_’s < 0.15). Neither downplaying nor amplifying the seriousness of the virus affected the subjects’ policy attitudes or behavioral intentions.

### Exploratory analyses

Implicit in Hypotheses 2–4 is the proposition that the effect of President Trump’s rhetoric is mediated by the perceived seriousness of the coronavirus’s death toll, because the treatment stimuli differ in the extent to which they downplay its seriousness (Gerber and Green 2012, chap. 5). Whether the president’s rhetoric operates through perceived seriousness can be examined more explicitly, however, by conducting exploratory mediation analysis. These exploratory analyses will be conducted to estimate the indirect effects of the Problem-Minimizing treatment on Trump’s job approval, support for social distancing policies, and intent to comply with social distancing.

To explore the mediating effect of perceived problem seriousness, I conducted a series of simple mediation analyses^3^ in which assignment-to-treatment is the explanatory variable; perceived seriousness of the coronavirus is the mediator; and evaluations of Trump, support for social distancing policies, and intentions to follow guidelines are the outcome variables. Political ideology and political interest are entered as covariates.

The indirect effects of President Trump’s problem-minimizing rhetoric on his performance evaluation through perceived seriousness of the virus are not significant (Hypothesis 2). The 98.75% confidence intervals contain 0. The same is true for the policy attitude and behavioral intention outcome variables (Hypothesis 4).

The Problem-Minimizing with Counterargument treatment, however, caused a strong (backfiring) effect, decreasing the perceived seriousness of the death toll by 7.4% points (*p* < 0.001, two-tailed; *BF*_10_ = 0.298). This result was not anticipated in the pre-analysis plan, but it highlights the increasingly limited capacity for counter-attitudinal facts to help partisans hold their leaders accountable (Agadjanian 2020). The backfiring effect was mediated by the seriousness with which subjects regarded the death toll (Indirect effect = 0.011, Boostrapped CI = [0.005, 0.019]). The positive indirect effect arises because it is the product of two negative effects: the treatment reduced perceived seriousness and higher perceived seriousness is associated with lower pandemic-specific performance evaluations for the president. The indirect effect is also moderately robust to a violation of the sequential ignorability assumption (Imai et al. 2011): the effect loses significance at values of *ρ*, the sensitivity parameter, greater than −0.3.^4^

## Discussion

Hypothesis 1 – that President Trump’s rhetoric would sway Republicans’ perceptions of the seriousness of the coronavirus – received no direct support here. Because Republicans became considerably less concerned about the coronavirus between April and June (Hetherington and Mehlhaff 2020) – and this experiment was fielded in early June – it is likely that I failed to reject the null hypothesis because the subjects had already shifted their perceptions in response to the elite messaging. These firmly held opinions about the virus and the president were likely the reason that information critical of the Trump administration’s efforts to combat the virus was met with backlash. Subjects may have entrenched their pro-Trump evaluations because they were already persuaded that the virus’s death toll was not serious and that the president had done all that was necessary.^5^ I failed to anticipate that counter-attitudinal information would diminish concern about the virus, but it is indicative of a tendency for partisans to downplay the importance of their leaders’ errors (Bartels 2002; Gaines et al. 2007), and to follow their interpretation of events (Bisgaard and Slothuus 2018a).

National approval of President Trump’s pandemic-specific job performance was also fairly crystallized by this time, as it remained largely unchanged between late May and late July in spite of growing infection rates in much of the country (Baum et al. 2020). Thus, direct evidence in favor of Hypothesis 2 was not found: approval of Trump’s job performance was no different between the Problem-Minimizing condition and control. Hypothesis 3 – that criticizing the president’s pre-pandemic preparation would lower his performance evaluation – received no support. In fact, the Problem-Minimizing with Counterargument treatment caused another backfiring effect in which participants *increased* their approval of the president. This too is suggestive of a Republican electorate already firmly believing in the president’s good performance. Similarly, their policy attitudes and intentions regarding social distancing were unmoved, lending no support for Hypothesis 4.

## Conclusion

By many accounts, President Trump and his administration mishandled the federal government’s response to the coronavirus pandemic. Nevertheless, throughout the process, the administration sent mixed messages about the severity of the virus while also praising the job they had been doing, particularly in the early months during the daily task force news briefings, but even through to the presidential debates. This paper sought to test a suite of hypotheses around the idea that President Trump’s arguments affected his supporters’ views of the virus’s seriousness for public health, the job he’s done, support for social distancing mandates, and intention to follow social distancing guidelines.

Given the unusually high salience of the pandemic, the null results of the experiment are not altogether surprising. It is noteworthy, however, that once their party’s leader had established how (un)seriously they should regard over 100,000 US deaths, challenging the president’s narrative with historical information about his policy decisions rallied followers around his interpretation of the facts even more.

## Footnotes

1 Support for this research was provided by the Texas Politics Project at the University of Texas at Austin. The data, code, and any additional materials required to replicate all analyses in this article are available at the *Journal of Experimental Political Science Dataverse* within the Harvard Dataverse Network, at: doi: 10.7910/DVN/JXPS9T. I report no conflicts of interest in the writing of this paper.

2 Bayes factors are calculated using the BayesFactor package in R and are subscripted 10 to indicate the ratio of the alternative hypothesis, *H*_1_, to the null, *H*_0_.

3 Analyses were conducted in R using the PROCESS 3.5 package provided by Hayes (Hayes 2018).

4 The omitted confounder would need to have correlate more strongly with the focal variables than ideological identification and approval of Trump’s pandemic job performance (*r* = 0.34).

5 It is possible that the backlash occurred because attitudes about the president’s overall performance in office are already crystallized, and that any critique of his decision-making would evoke similar a backlash.

## Supplementary material

For supplementary material accompanying this paper visit https://doi.org/10.1017/XPS.2020.45. click here to view supplementary material
