# Supplementary material for: How Bad is it? Elite Influence and the Perceived Seriousness of the Coronavirus Pandemic
Source: Journal of Experimental Political Science. 2020 Dec 18:1–9. doi: 10.1017/XPS.2020.45 (PMC7870919; doi:10.1017/XPS.2020.45)
Supplement: Supplementary file 1 [file S2052263020000457sup.zip › S2052263020000457sup001.pdf]

Supplementary Materials

Table 1 – Descriptive Statistics by Experimental Condition

Condition	Age	Ideology	Pol. Interest
Control	37 (13.2)	5.29 (1.17)	3.64 (0.99)
Minimize	36.65 (13.7)	5.22 (1.26)	3.7 (1.00)
Minimize + Counter-Info	37.41 (14.5)	5.32 (1.24)	3.63 (1.00)
Amplify	37.69 (13.8)	5.33 (1.18)	3.67 (0.97)

Note: Means with standard deviations in parentheses.

Table 2 – Outcome Variable Means, SDs of Differences in Means, and Ns

Conditions	Problem Seriousness	Trump Job Eval.	Soc. Distancing Policy Support	Soc. Distancing Intention
Control	0.69	0.6	0.64	0.50
	N = 424	N = 421	N = 421	N = 418
Minimize	0.67	0.63^{+}	0.65	0.50
	[-0.03, 0.06]	[-0.09, 0.01]	[-0.05, 0.04]	[-0.05, 0.05]
	N = 407	N = 405	N = 403	N = 402
Amplify	0.67	0.61	0.63	0.50
	[-0.02, 0.07]	[-0.07, 0.03]	[-0.03, 0.06]	[-0.05, 0.05]
	N = 398	N = 397	N = 397	N = 392
Minimize + Counter Info	0.61^{*}	0.64^{*}	0.62	0.52
	[0.03, 0.12]	[-0.1, 0.002]	[-0.02, 0.07]	[-0.07, 0.02]
	N = 386	N = 384	N = 382	N = 385

Note: Cells contain mean values of the dependent variables scaled from 0–1. Larger values indicate higher seriousness, better evaluation, and stronger support/intention. Brackets contain 98.75% confidence intervals of the difference in means between control and treatment condition. $^{+}p < 0.05$, $^{*}p < 0.0125$, one-tailed. Observations were dropped only if they had missing data on the outcome variable.

Table 3 – Trump Job Performance Scale Information

Item	Mean	SD
Good-Poor	0.62	0.28
Competent-Incompetent	0.62	0.29
Effective-Ineffective	0.61	0.28
Helpful-Unhelpful	0.62	0.28

Note: All item prompts read: "How would you characterize the job that the Trump administration has done to limit the number of deaths from COVID-19?" Originally measured on a 9-point scale, here rescaled 0-1. Higher values indicate a better job performance evaluation.

Table 4 – Policy Support Scale Information

Item	Mean	SD
Requiring most businesses other than groceries and pharmacies to close	0.55	0.29
Asking people to avoid gathering in groups more than ten	0.71	0.27
Restricting international travel to the U.S.	0.84	0.20
Asking people to shelter in place and not leave their homes except for essential activities	0.66	0.29
Canceling major sports and entertainment events	0.71	0.27
Limiting restaurants to carryout only	0.68	0.28
Closing K-12 schools	0.69	0.28
Permitting anyone to vote by mail if they want to	0.63	0.33

Note: All item prompts read: "Thinking about some steps that have been announced in some areas to address the coronavirus outbreak, in general do you support or oppose each of the following?" Originally a 7-point "strongly support" to "strongly oppose" response scale, rescaled here 0-1. Higher values indicate stronger support.

Table 5 – Behavioral Intention Scale Information

Item	Mean	SD
Go to a shopping mall	0.57	0.30
Go to dinner at a friend's house	0.76	0.27
Fly on an airplane	0.48	0.30
Eat at a restaurant	0.66	0.30
Wear a face mask in public (reverse-coded)	0.44	0.31
Attend a wedding reception	0.59	0.31
Go to the movies	0.49	0.31
Go to church services	0.58	0.33

Note: All item prompts read: "If restrictions were (or are) lifted on the advice of public health officials to do the following, how likely would you be (or are you) to:" Originally a 7-point "extremely likely" to "extremely unlikely" response scale, rescaled here 0-1. Higher values indicate greater likelihood.

Table 6 – Experimental Stimuli

Item	Text
Control	The death toll from COVID-19 in the United States has passed 100,000, just three months after the U.S. recorded its first deaths from the novel coronavirus that emerged in China late last year.
Minimize	On the U.S. death toll from COVID-19, President Trump said: "If I hadn't done my job well, and early, we would have lost 1 and a half to 2 million people, as opposed to the 100,000 plus that looks like will be the number. That's 15 to 20 times more than we will lose. I shut down entry from China very early!"
Amplify	On the U.S. death toll from COVID-19, President Trump said: "It's something that nobody could have ever projected. It's been over 100 years that a thing like this has happened. It's just a very tough situation for the people of our country. All the loss, the death. It's a terrible thing."
Minimize + Counter Info	Minimize text plus<br>Some observers have tried to put the death toll and the government response in context. For example, in May 2018, the Trump administration dissolved a White House pandemic task force that was charged with preparing the U.S. government for global diseases like COVID-19. The office would have coordinated the federal government's response by identifying needs among state and local officials, facilitating communication between them and scientific experts, and elevating urgent issues fast, so they didn't linger or devolve to inaction, as with coronavirus testing in the United States.

Note: Control text is taken from a May 28, 2020 DemocracyNow.org headline. President Trump's quotation comes from a Twitter post he made on May 26, 2020. The counterargument material on the pandemic task force is excerpted from a *Washington Post* opinion article: https://www.washingtonpost.com/outlook/nsc-pandemic-office-trump-closed/2020/03/13/a70de09c-6491-11ea-acca-80c22bbee96f_story.html.

Compliance with Reporting Standards

Hypotheses

The paper tests four hypotheses: first, that Trump's problem-minimizing rhetoric lowers how serious the death toll of the coronavirus seems; second, that Trump's problem-minimizing or problem-amplifying rhetoric increases his job approval among Republican identifiers; third, that providing evidence showing that President Trump diminished the government's preparedness for a pandemic would lower his performance evaluation; and fourth, that the president's problem-minimizing (problem-amplifying) rhetoric would increase (decrease) Republicans' support for social distancing policy and intentions to socially distance.

Subjects and Context

Republican-identifying U.S.-based adults were the subjects selected for the experiment and surveyed between June 4 and June 9, 2020. The subjects were recruited through the online survey platform Prolific and were screened prior to taking the survey using Prolific's built-in screening functions. Prolific recruits its participants primarily through posts on social media platforms, poster/flyer campaigns on universities, and through referrals from researchers and participants already using the site.

Allocation Method

The treatments were randomized using Qualtrics's built-in randomization tool. Subjects were placed in one of the four conditions with 1/4 probability. Table 1 in the appendix, above, reports means and standard deviations of demographic covariates to support the success of the randomization.

Treatments

Exact wording of the experimental stimuli are presented in table 6 in the appendix, above. The full questionnaire can be found at the Dataverse repository page.

Results

The treatment-indicator variable was formed by recoding the randomization-indicator variable provided by the Qualtrics software. Subjects assigned to the control group were given a value of 0; they were given a 1 if assigned to the Problem-Minimizing condition; a 2 if assigned to the Problem-Minimizing with Counterargument condition; and a 3 if assigned to the Problem-Amplifying condition.

Four outcome variables were used: perceived seriousness of the coronavirus death toll, evaluation of President Trump's pandemic-specific job performance, support for social-distancing policies, and intentions to engage in risky health-related behaviors. The exact wording of all items can be found in appendix tables 2-4 above. All outcome variables were constructed to take values between 0 and 1. The three scales were then formed by taking the average of all their constituent items. No items were dropped because of low reliability.

Missing Data

A very trivial number of respondents were dropped from the analyses due to missing data on the outcome variables. None were dropped for the perceived seriousness analyses; a maximum of 4 were dropped from the Trump performance evaluation analyses; a maximum of 4 were dropped from the social distancing policy support analyses; and a maximum of 6 were dropped from the behavioral intention analyses.

Other Information

The study was approved by University of Texas at Austin IRB under protocol 2020-05-0041. It was preregistered at https://osf.io/kjh9b. Funding was provided by the Texas Politics Project, which is housed at the University of Texas at Austin. The Texas Politics Project did not help conduct any of the analyses in the study, and it placed no restrictions on what findings could be published. The replication dataset can be found at https://doi.org/10.7910/DVN/JXPS9T.
