# Supplementary material for: How Bad is it? Elite Influence and the Perceived Seriousness of the Coronavirus Pandemic
Source: Journal of Experimental Political Science. 2020 Dec 18:1–9. doi: 10.1017/XPS.2020.45 (PMC7870919; doi:10.1017/XPS.2020.45)
Supplement: Supplementary file 1 [file S2052263020000457sup.zip › S2052263020000457sup002.docx]

COVID-19 & Accountability JEPS

Start of Block: Intro

consent Conducted by ~~~ You are being asked to participate in a research study.  This form provides you with information about the study. Participation in this study is voluntary and refusal to participate will involve no penalty or loss of benefits to which you are otherwise entitled.  Also, you may discontinue participation at any time without penalty or loss of benefits to which you are otherwise entitled.  The purpose of the study is to better understand people’s attitudes about the coronavirus pandemic. If you agree to participate, we will ask you to answer survey questions about the coronavirus pandemic. Total estimated time to participate in the study is 10 minutes.   
   
Risks / Benefits:  The risk associated with this study is no greater than everyday life.  There are no benefits for participation in this study.      Confidentiality and Privacy Protections:    The records of this study will be stored securely and the data will be anonymized. Authorized persons from ~~~ , members of the Institutional Review Board have the legal right to review your research records and will protect the confidentiality of those records to the extent permitted by law.  All publications will exclude any information that will make it possible to identify you as a subject. Further, confidentiality will be maintained by not asking you to provide any identifying information in the survey.  Contacts and Questions:  If you have questions later, want additional information, or wish to withdraw your participation please contact the researcher conducting the study. His name and e-mail address are at the top of this page. If you have questions about your rights as a research participant, complaints, concerns, or questions about the research please contact the Office of Research Support at ~~~ Please print out or request a copy of this page for your records.     Statement of Consent:     I have read the above information and have sufficient information to make a decision about participating in this study.  I consent to participate in the study.

- Yes (4)
- No (5)

| Page Break |  |
| --- | --- |

Q77 **The survey begins with a short newspaper clipping about the public health consequences of the coronavirus. Please read it and think about it before you proceed to the next page.**

End of Block: Intro

Start of Block: Manipulations

minimize On the U.S. death toll from COVID-19, President Trump said: "If I hadn't done my job well, and early, we would have lost 1 and a half to 2 million people, as opposed to the 100,000 plus that looks like will be the number. That's 15 to 20 times more than we will lose. I shut down entry from China very early!"

amplify On the U.S. death toll from COVID-19, President Trump said: "It’s something that nobody could have ever projected. It’s been over 100 years that a thing like this has happened. It's just a very tough situation for the people of our country. All the loss, the death. It's a terrible thing.''

minimize.context On the U.S. death toll from COVID-19, President Trump said: "If I hadn't done my job well, and early, we would have lost 1 and a half to 2 million people, as opposed to the 100,000 plus that looks like will be the number. That's 15 to 20 times more than we will lose. I shut down entry from China very early!"

Some observers have tried to put the death toll and the government response in context. For example, in May 2018, the Trump administration dissolved a White House pandemic task force that was charged with preparing the U.S. government for global diseases like COVID-19. The office would have coordinated the federal government’s response by identifying needs among state and local officials, facilitating communication between them and scientific experts, and elevating urgent issues fast, so they didn’t linger or devolve to inaction, as with coronavirus testing in the United States.

control The death toll from COVID-19 in the United States has passed 100,000, just three months after the U.S. recorded its first deaths from the novel coronavirus that emerged in China late last year.

End of Block: Manipulations

Start of Block: Problem Status

deaths.problem How big of a problem do you think that the projected total U.S. death toll from COVID-19 is?

- A very big problem (1)
- A big problem (2)
- Somewhat of a problem (3)
- Not a big problem (4)
- No problem at all (5)

End of Block: Problem Status

Start of Block: Government Performance

Q49 Many leaders across society are responding to the coronavirus pandemic. Here we ask you to think specifically about how President Trump and his administration have done with respect to the public health.

trump.incomp How would you characterize the job that the Trump administration has done to limit the number of deaths from COVID-19?

- Extremely competent (1)
- 2 (2)
- 3 (3)
- 4 (4)
- 5 (5)
- 6 (6)
- 7 (7)
- 8 (8)
- Extremely incompetent (9)

trump.weak

- Extremely good (1)
- 2 (2)
- 3 (3)
- 4 (4)
- 5 (5)
- 6 (6)
- 7 (7)
- 8 (8)
- Extremely poor (9)

trump.helpful

- Extremely helpful (1)
- 2 (2)
- 3 (3)
- 4 (4)
- 5 (5)
- 6 (6)
- 7 (7)
- 8 (8)
- Extremely unhelpful (9)

trump.effective

- Extremely effective (1)
- 2 (2)
- 3 (3)
- 4 (4)
- 5 (5)
- 6 (6)
- 7 (7)
- 8 (8)
- Extremely ineffective (9)

End of Block: Government Performance

Start of Block: Manipulation Check

Display This Question:

If The death toll from COVID-19 in the United States has passed 100,000, just three months after the... Is Displayed

| 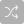 |
| --- |

man.check Who of the following were NOT quoted in the newspaper clipping you read earlier? Check all that apply.

- Donald Trump (1)
- Anthony Fauci (2)
- Mitch McConnell (3)
- Mike Pence (4)

End of Block: Manipulation Check

Start of Block: Distancing Policy Attitudes

Q80 Thinking about some steps that have been announced in some areas to address the coronavirus outbreak, in general do you support or oppose each of the following?

| 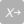 |
| --- |

Q47  Requiring most businesses other than groceries and pharmacies to close

- Strongly oppose (7)
- Oppose (6)
- Somewhat oppose (5)
- Neither support nor oppose (4)
- Somewhat support (3)
- Support (2)
- Strongly support (1)

| 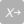 |
| --- |

Q60 Asking people to avoid gathering in groups more than ten

- Strongly oppose (7)
- Oppose (6)
- Somewhat oppose (5)
- Neither support nor oppose (4)
- Somewhat support (3)
- Support (2)
- Strongly support (1)

| 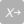 |
| --- |

Q61 Restricting international travel to the U.S.

- Strongly oppose (7)
- Oppose (6)
- Somewhat oppose (5)
- Neither support nor oppose (4)
- Somewhat support (3)
- Support (2)
- Strongly support (1)

| 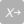 |
| --- |

Q62 Asking people to shelter in place and not leave their homes except for essential activities

- Strongly oppose (7)
- Oppose (6)
- Somewhat oppose (5)
- Neither support nor oppose (4)
- Somewhat support (3)
- Support (2)
- Strongly support (1)

| 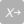 |
| --- |

Q63 Canceling major sports and entertainment events

- Strongly oppose (7)
- Oppose (6)
- Somewhat oppose (5)
- Neither support nor oppose (4)
- Somewhat support (3)
- Support (2)
- Strongly support (1)

| 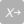 |
| --- |

Q64 Limiting restaurants to carryout only

- Strongly oppose (7)
- Oppose (6)
- Somewhat oppose (5)
- Neither support nor oppose (4)
- Somewhat support (3)
- Support (2)
- Strongly support (1)

| 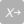 |
| --- |

Q65 Closing K-12 schools

- Strongly oppose (7)
- Oppose (6)
- Somewhat oppose (5)
- Neither support nor oppose (4)
- Somewhat support (3)
- Support (2)
- Strongly support (1)

| 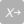 |
| --- |

Q66 Permitting anyone to vote by mail if they want to

- Strongly oppose (7)
- Oppose (6)
- Somewhat oppose (5)
- Neither support nor oppose (4)
- Somewhat support (3)
- Support (2)
- Strongly support (1)

End of Block: Distancing Policy Attitudes

Start of Block: Distancing Intentions

Q65 If restrictions were (or are) lifted on the advice of public health officials to do the following, how likely would you be (or are you) to:

| 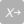 |
| --- |

Q67 Go to a shopping mall

- Extremely likely (7)
- Moderately likely (6)
- Slightly likely (5)
- Neither likely nor unlikely (4)
- Slightly unlikely (3)
- Moderately unlikely (2)
- Extremely unlikely (1)

| 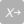 |
| --- |

Q77 Go to dinner at a friend's house

- Extremely likely (7)
- Moderately likely (6)
- Slightly likely (5)
- Neither likely nor unlikely (4)
- Slightly unlikely (3)
- Moderately unlikely (2)
- Extremely unlikely (1)

| 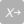 |
| --- |

Q76 Fly on an airplane

- Extremely likely (7)
- Moderately likely (6)
- Slightly likely (5)
- Neither likely nor unlikely (4)
- Slightly unlikely (3)
- Moderately unlikely (2)
- Extremely unlikely (1)

| 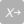 |
| --- |

Q72 Eat at a restaurant

- Extremely likely (7)
- Moderately likely (6)
- Slightly likely (5)
- Neither likely nor unlikely (4)
- Slightly unlikely (3)
- Moderately unlikely (2)
- Extremely unlikely (1)

| 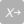 |
| --- |

Q75 Wear a face mask in public

- Extremely likely (7)
- Moderately likely (6)
- Slightly likely (5)
- Neither likely nor unlikely (4)
- Slightly unlikely (3)
- Moderately unlikely (2)
- Extremely unlikely (1)

| 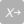 |
| --- |

Q73 Attend a wedding reception

- Extremely likely (7)
- Moderately likely (6)
- Slightly likely (5)
- Neither likely nor unlikely (4)
- Slightly unlikely (3)
- Moderately unlikely (2)
- Extremely unlikely (1)

| 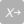 |
| --- |

Q74 Go to the movies

- Extremely likely (7)
- Moderately likely (6)
- Slightly likely (5)
- Neither likely nor unlikely (4)
- Slightly unlikely (3)
- Moderately unlikely (2)
- Extremely unlikely (1)

| 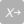 |
| --- |

Q69 Go to church services

- Extremely likely (7)
- Moderately likely (6)
- Slightly likely (5)
- Neither likely nor unlikely (4)
- Slightly unlikely (3)
- Moderately unlikely (2)
- Extremely unlikely (1)

End of Block: Distancing Intentions

Start of Block: Pandemic Knowledge

Q49 Please answer the following questions to the best of your ability. It's alright if you don't know the answer.

Display This Question:

If The death toll from COVID-19 in the United States has passed 100,000, just three months after the... Is Displayed

Or Or On the U.S. death toll from COVID-19, President Trump said: "It’s something that nobody could hav... Is Displayed

pretreated President Trump has suggested on a few occasions that the death toll from COVID-19 in the U.S. could have been much higher, between 1.5 and 2.2 million people, had no social distancing guidelines been put in place. Have you heard the president make this argument before?

- Yes, several times (1)
- Yes, once (2)
- No (3)
- Not sure (4)

| 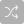 |
| --- |

animal.origin What animal do coronaviruses typically come from?

- Bats (1)
- Rats (2)
- Fish (3)
- Mosquitos (4)
- Don't know (5)

| 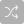 |
| --- |

hydroxy What malaria medication did President Trump suggest as a treatment for COVID-19?

- Hydroxycloroquine (1)
- Novaferon (2)
- Bromhexine (3)
- Dihydroartemisinin (4)
- Don't know (5)

| 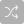 |
| --- |

who.hhs Who is the current Secretary of Health and Human Services?

- Alex Azar (4)
- Mark Esper (5)
- Steven Mnuchin (9)
- Mike Pompeo (6)
- Don't know (8)

ppe For doctors and nurses, what does "PPE" stand for?

________________________________________________________________

End of Block: Pandemic Knowledge

Start of Block: Extra

trump.likely Has Trump's handling of the pandemic with regard to public health changed how likely you are to vote for him in November?

- Extremely more likely (1)
- Moderately more likely (2)
- Slightly more likely (3)
- Neither more likely nor less likely (4)
- Slightly more unlikely (5)
- Moderately more unlikely (6)
- Extremely more unlikely (7)

trump.save To what extent do you agree with the following statement: President Trump could have saved tens of thousands of lives had he issued social distancing guidelines sooner.

- Strongly agree (4)
- Agree (5)
- Somewhat agree (6)
- Neither agree nor disagree (7)
- Somewhat disagree (8)
- Disagree (9)
- Strongly disagree (10)

trump.taskforce Had President Trump not dissolved his pandemic task force in 2018, would his administration's response to COVID-19 have been better?

- Much better (11)
- Moderately better (12)
- Slightly better (13)
- About the same (14)
- Slightly worse (15)
- Moderately worse (16)
- Much worse (17)

End of Block: Extra

Start of Block: Demographics

Q26 Generally speaking, do you think of yourself as a Republican, a Democrat, an Independent, or what?

- Republican (1)
- Democrat (2)
- Independent (3)
- Other (4) ________________________________________________

Display This Question:

If Generally speaking, do you think of yourself as a Republican, a Democrat, an Independent, or what? = 3

Q36 Do you think of yourself as closer to the Republican Party or to the Democratic party?

- Republican Party (1)
- Democratic Party (2)
- Neither (3)

Display This Question:

If Generally speaking, do you think of yourself as a Republican, a Democrat, an Independent, or what? = 1

Or Generally speaking, do you think of yourself as a Republican, a Democrat, an Independent, or what? = 2

Q30 Would you consider yourself a strong ${Q26/ChoiceGroup/SelectedChoices} or not very strong ${Q26/ChoiceGroup/SelectedChoices}?

- Strong (1)
- Not very strong (2)

ideology Generally speaking, how would you describe your own political point of view?

- Very liberal (1)
- Liberal (2)
- Somewhat liberal (3)
- Moderate (4)
- Somewhat conservative (5)
- Conservative (6)
- Very conservative (7)

| 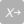 |
| --- |

follow.news How closely have you been following the news about the outbreak of the coronavirus strain known as Covid-19?

- Very closely (5)
- Closely (4)
- Somewhat closely (3)
- Not too closely (2)
- Not at all closely (1)

| 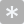 |
| --- |

state What state do you currently live in? (Please give two-letter abbreviation.)

________________________________________________________________

gender What is your gender?

- Male (1)
- Female (2)
- Non-binary (5)

| 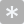 |
| --- |

age What is your age?

________________________________________________________________

End of Block: Demographics

Start of Block: Debrief

debrief **Debriefing Form** **Title: How Bad Is It?: Elite Influence and the Perceived Seriousness of the Coronavirus Pandemic** **Purpose of the Study**In the informed consent document you read and signed before the study started, you were told that the purpose of the study was to get a sense of how people are thinking about the public health consequences of the pandemic and the role that political leaders play in dealing with the problem. The real purpose of this study is to see if President Trump’s messaging that downplays the seriousness of the death toll of the virus decreases people’s sense of its seriousness. You were not given the real purpose of the study in case that would have changed your answers or how you acted. This is an ongoing study and we do not want this detail to influence future participants. We ask that you do not tell others about the real purpose until the study is over.  **Permission to Use Data**Now that you have been told the real purpose of this study, we want to make sure that you understand the real purpose of the study and ask for your permission to use your data.  Remember, we want to understand how people act in general. We will never draw any results about you personally.   If you do not want your data included in this study, your data will be immediately destroyed and it will not be analyzed or included as part of the study report. Your refusal will not impact current or future relationships with ~~~.  It will also not affect the compensation you were promised at the start of the study. If you want more information about this study, you can talk to any of the investigators: ~~~  If you would like to talk about this study with someone not involved in the study, you can contact The Office of Research Support at ~~~ **Consent**Your selection below means that you understand the real purpose of the study. Your selection does not mean you are waiving any legal rights. Please indicate if you do or do not agree for us to use your data now that you know the real purpose of the study.

- I understand the real purpose of the study and allow the researchers to use my data. (1)
- I understand the real purpose of the study and do NOT allow the researchers to use my data. (2)

End of Block: Debrief

Start of Block: Survey Feedback

| 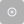 |
| --- |

prolific.id Please enter your Prolific ID here

________________________________________________________________

feedback What did you think of the survey? Leave any comments here. Thank you!

________________________________________________________________

________________________________________________________________

________________________________________________________________

________________________________________________________________

________________________________________________________________

End of Block: Survey Feedback
